# Evaluation and Immunogenicity of Combined Liposome-Based Vaccine Candidates against Hepatitis E and B Viruses in Rhesus Monkeys

**DOI:** 10.3390/vaccines12010053

**Published:** 2024-01-05

**Authors:** Tejaswini Deshmukh, Rachita Shah, Pradip Devhare, Kavita Lole, Vidya Arankalle

**Affiliations:** 1 Hepatitis Group, ICMR-National Institute of Virology, 130/1, Pune 411021, India; deshmukh.t@gov.in (T.D.); shahrachita20@gmail.com (R.S.); pradip.devhare@velsera.com (P.D.); lole.k@gov.in (K.L.); 2 1404 H1 Kumar Pruthvi, Kondhwa Budruk, Pune 411048, India; 3 Velsera, Pune 411016, India; 4 Interactive Research School for Health Affairs, Bharati Vidyapeeth University, Pune 411043, India

**Keywords:** hepatitis E, hepatitis B, neutralizing epitope/s, small envelope, vaccine candidates, liposomes, rhesus monkeys

## Abstract

The administration of vaccines using a combination approach ensures better coverage and reduces the number of injections and cost. The present study assessed liposome-complexed DNA-corresponding proteins of hepatitis E and B viruses (HEV and HBV) as combined vaccine candidates in rhesus monkeys. The HEV and HBV components consisted of 450 bps, neutralizing the epitope/s (NE) region, and 685 bps small (S) envelope gene-corresponding proteins, respectively. Three groups (*n* = 2 monkeys/group) were intramuscularly immunized with a total of three doses of NE Protein (Lipo-NE-P), NE DNA + Protein (Lipo-NE-DP), and each of NE and S DNA + Protein (Lipo-NES-DP), respectively, given one month apart. All immunized monkeys were challenged with 10,000 fifty percent monkey infectious dose of homologous HEV strain. Post-immunization anti-HEV antibody levels in monkeys were 59.4 and 148.4 IU/mL (Lipo-NE-P), 177.0 and 240.8 IU/mL (Lipo-NE-DP), and 240.7 and 164.9 IU/mL (Lipo-NES-DP). Anti-HBV antibody levels in Lipo-NES-DP immunized monkeys were 58,786 and 6213 mIU/mL. None of the challenged monkeys showed viremia and elevation in serum alanine amino transferase levels. Monkeys immunized with Lipo-NE-DP and Lipo-NES-DP exhibited a sterilizing immunity, indicating complete protection, whereas monkeys immunized with Lipo-NE-P showed limited viral replication. In conclusion, the liposome-complexed DNA-corresponding proteins of HEV and HBV induced protective humoral immune responses to both components in monkeys and are worth exploring further.

## 1. Introduction

Enterically transmitted hepatitis A and E viruses (HAV and HEV) cause self-limiting acute viral hepatitis (AVH), occasionally leading to acute liver failure (ALF). Epidemics of hepatitis E have continued to occur since its first large recognized epidemic, initially thought to be associated with HAV and subsequently shown to be due to enterically transmitted non-A non-B (ET-NANB) agent, finally designated as HEV [1]. Subsequently, HEV was shown to be frequently associated with waterborne epidemics as well as sporadic cases of acute hepatitis [2]. Rarely, HEV can cause chronic infections in immune-suppressed/-compromised individuals [3]. However, when it comes to pregnant women, HEV can progress to severe disease and mortality, especially in the second and third trimesters [4]. This high-risk population needs to be protected either by immunization or effective therapies. In the absence of an effective specific treatment, the management of symptoms remains the current strategy. Super-infection with HEV is responsible for 10–15% of cases of acute chronic liver failure (ACLF) in India. In the absence of liver transplantation, ACLF accounts for 50–60% mortality [4,5,6]. Thus, another high-risk group includes HBV and HCV carriers that develop severe disease when superinfected with HEV [4,5,6]. The national HBV and HCV rates have not been estimated recently. Even if we consider the 1% HBsAg and 0.2% HCV carrier rates, the numbers requiring vaccines will be high for the 1.4 billion population. It is also important to consider tribal populations with high carrier rates of HBV, HCV, or both. The successful development of recombinant protein-based HEV vaccines were immediately reported after the genomes were sequenced [7]. However, none of these became commercially available. HEV 239 or Hecolin manufactured by Xiamen Innovax Biotech Co. Ltd. (Fujian, China) was the first vaccine to be licensed in December 2011 in China but is not yet WHO pre-qualified and widely available [8]. This vaccine was licensed in Pakistan in 2020 [2]. A clinical trial (CTRI/2020/03/023769, Cadila Healthcare, Ahmedabad, India, May 2021) of a recombinant hepatitis E vaccine candidate has been registered in India [9]. Our group has worked extensively on the development of hepatitis E and B vaccine candidates using monovalent and combined approaches [10,11,12].

Safe and effective monovalent and combined vaccines in different formulations have been licensed against HAV and HBV and used globally for several decades [13,14,15]. Although used since the early 1980s, hepatitis B immunization was introduced in the national immunization program of India very late (2012) and was administered at birth and subsequently as part of three doses of the pentavalent vaccine (DPT + HepB + Hib) (UIP, India). For high-risk pregnant women, the hepatitis B vaccine is recommended [16]. Pregnancy is not contraindicated for the HBV vaccine [17]. Thus, our goal has been to develop an effective vaccine for hepatitis E that can be used during pregnancy and for HBV and HCV carriers. Given the requirement for an HBV vaccine for high-risk pregnant women, we decided to evaluate the HEV + HBV vaccine for this special category. With the controversial reports of rapid decline in anti-HEV titers following natural infection, we explored several approaches in mice and then proceeded to evaluate the vaccine candidates in rhesus monkeys [9,10,11], present study]. We assessed the immunogenicity of ORF2 and NE-based hepatitis E vaccine candidates in mice employing different approaches such as DNA, rProtein, DNA-Prime-Protein-Boost, and DNA + corresponding rProtein using alum and liposomes as adjuvants as monovalent candidates or in combination with the HBV component (DNA and/or protein forms of a small surface region, S). Due to strict national restrictions on the use of monkeys, we could not include additional, desired groups. Our present study reports the humoral immune response induced in rhesus monkeys immunized with liposome formulations of (1) rNE Protein (Lipo-NE-P); (2) rNE DNA + corresponding Protein (Lipo-NE-DP); and (3) rNE and rS DNAs + corresponding Proteins of HEV and HBV, respectively (Lipo-NES-DP).

## 2. Materials and Methods

### 2.1. Ethics Statement

This study was approved by the Institutional and National Ethics Committees for the Purpose of Control and Supervision of Experiments on Animals (CPCSEA) F.No.25/11/2005-AWD. Housing, maintenance, and care of rhesus monkeys complied with the guidelines and requirements of CPCSEA.

### 2.2. Primates

Ten anti-HEV and anti-HBV antibody-negative female rhesus monkeys (*Macaca mullata*) of about two years of age were used.

### 2.3. Candidate Vaccines

#### 2.3.1. Construction of Recombinant DNA Plasmids

The NE (450 bps) and S (685 bps) genes of HEV and HBV, respectively, were cloned in the pVAX1 vector and prepared according to the procedures described previously [11,12]. The pVAX1 vector, 3.0 kbs (Invitrogen, Carlsbad, CA, USA), was approved for human use as a DNA vaccine by the U. S. Food and Drug Administration. Plasmids were purified using endo-free plasmid Maxi-prep columns (Qiagen, Hilden, Germany). Plasmid quality was tested by agarose gel electrophoresis and purity and quantity were assessed by spectrophotometry. For experimental use, the plasmid DNA was reconstituted in endotoxin-free water at a concentration of 1.0 µg/µL.

#### 2.3.2. Recombinant Proteins

The gene for the NE protein (150 a.a.) was cloned in the pET-15b vector (5708 bps, Novagen, Germany) and expressed in *Escherichia coli* strain BL21(DE3)pLysS with a 6X Histidine tag at its amino-terminal. The NE protein was purified using Nickel-chelated resin (ProBond, Invitrogen, Life Technologies, Carlsbad, CA, USA) as described earlier [12]. Endotoxin levels in the final protein formulations were tested using the Limulus Amebocyte Lysate (LAL) test (Pierce LAL Chromogenic Endotoxin Quantitation Kit; Thermo Fisher Scientific, Waltham, MA, USA) and were <10 EU/mg of protein. Purified S protein (226 a.a.) expressed in yeast cells was obtained from the hepatitis B vaccine manufacturer, Serum Institute of India Private Ltd. (SIIPL), Pune, Maharashtra, India [11].

#### 2.3.3. Liposome Preparations

DNAs and/or corresponding proteins (NE and/or S) were complexed with cationic liposomes prepared using hydrogenated soy phosphatidyl choline (Avanti polar lipids, Alabaster, AL, USA), cholesterol, and stearylamine (Sigma, St. Louis, MO, USA) using the dehydration and rehydration method. The antigen:liposome mass ratio was 1:200 (*w*/*w*). The mixture was lyophilized and reconstituted overnight in sterile 10 mM phosphate buffer saline, pH 7.2 (PBS) before use [11].

### 2.4. Monkey Immunization

A total three doses of each vaccine candidate were administered intramuscularly (thigh muscles) at 0, 4, and 8 weeks to individual monkeys assigned to three separate immunization groups. Each of the 0.5 mL of liposome dose formulations contained 20 µg DNA and/or 20 µg protein of NE and/or S components in sterile PBS. Table 1 provides the details of the different vaccine candidate formulations evaluated in groups of monkeys.

### 2.5. Challenge of Immunized Monkeys

Immunized monkeys were challenged with the homologous HEV strain (PM2000 strain, genotype 1 HEV) described earlier (Table 1). The infectivity titer of 10% (*w*/*v*) fecal suspension was estimated to be 10^6^ fifty percent monkey infectious dose (MID_50_) per gram of feces in the earlier titration experiment [10]. The 10% (*w*/*v*) fecal suspension contained 10^7^ HEV RNA copies/mL in quantitative real-time PCR (qRT-PCR). Each of the immunized monkeys (MM 407, 408, 417, 419, 420, and 423) were challenged intravenously two weeks after the last dose with 1 mL of 1:10 diluted 10% (*w*/*v*) fecal suspension (10^6^ HEV RNA copies; 10,000 MID_50_).

Similarly, four unimmunized monkeys (MM 421, 422, 427, and 428) were inoculated intravenously with 1 mL of 1:10 diluted 10% (*w*/*v*) fecal suspension (10^6^ HEV RNA copies; 10,000 MID_50_) at the same time point and served as the control group.

### 2.6. Monitoring of Monkeys

For monitoring HEV infection, three parameters were used: (1) serum alanine aminotransferase (ALT) evidencing hepatitis; (2) detection of HEV RNA in feces and blood; and (3) seroconversion to anti-HEV antibodies. The ALT levels in monkey sera were estimated using a commercial kit (Span Diagnostics, India) following the manufacturer’s instructions. For defining the normal range of serum ALT in rhesus monkeys, serum samples collected from all the monkeys twice a week for 4 weeks before immunization/experimental infection were tested. The biochemical evidence of hepatitis was defined as a ≥ two-fold increase in the peak post-challenge/experimental infection when compared to pre-challenge/experimental infection ALT values. Following each immunization dose, monkeys were bled every 15 days and twice weekly up to 2 months post-challenge/experimental infection. Following HEV challenge/experimental infection, the fecal samples were collected twice daily for up to 2 months and stored immediately at −70 °C until they were used as 10% (*w*/*v*) suspensions in sterile PBS.

### 2.7. Serological Assays

Monkey serum samples were tested for anti-HEV antibodies using ORF2 and NE protein-based ELISAs as described previously [12]. To differentiate between vaccine/vaccine candidate- and natural/experimental HEV infection-induced antibody responses, an ELISA based on bacterially expressed N-ORF2 protein (not present in the vaccine candidates) was used as described previously [10]. Cut-off values for all three ELISAs (ORF2, NE, and N-ORF2) were calculated as three times the mean OD_492 nm_ values for the three pre-immunization/experimental infection monkey serum samples. The monkey serum samples showing OD_492 nm_ values ≥ cut-off values were considered reactive for antibodies to the respective antigens. The two-fold serum dilutions were tested in ORF2 and NE ELISAs. The reciprocal of the highest serum dilution with an OD_492 nm_ value ≥ ELISA cut-off value was noted as an anti-ORF2/NE antibody titer. Anti-HEV antibody levels were determined using ORF2 ELISA against the WHO reference reagent (National Institute for Biological Standards and Control, UK, catalogue no. 95/584) in immunized monkeys [18]. For the detection and quantitation of anti-HBV antibodies in the monkey serum samples, the commercially available chemiluminescent micro-particle immunoassay “ARCHITECT” Anti-HBs assay (Abbott, Ireland) was performed according to the manufacturer’s instructions.

Anti-HEV and/or HBV IgG subclass antibodies were detected in all the immunized monkeys just before and 4 weeks after the challenge using ORF2 ELISA, a procedure described previously [11,12]. Anti-HBV IgG subclass antibodies were detected using an ELISA procedure like that for anti-HEV IgG subclass antibody detection, except the coating was replaced by the S protein obtained from SIIPL. Anti-HEV IgG subclass antibodies were similarly detected in all the control monkeys 4 weeks after challenge using the ORF2 ELISA.

### 2.8. Molecular Assays

HEV RNA copies in the monkey fecal and serum samples were determined by a Taqman quantitative RT-PCR (q-RT-PCR) assay as described earlier [10].

### 2.9. Statistical Analyses

Statistical analyses were carried out using PASW Statistics version 18. For comparing anti-HEV antibody mean log_10_ titers between groups, at any time point, one-way ANOVA with Tukey’s post hoc test was conducted. For comparing the anti-HEV antibody mean log_10_ titers between any two time points for a particular group, a paired *t*-test was used. For comparing mean log_10_ viral load between the control and immunized group (Lipo-NE-P), all data for all days and all monkeys in the corresponding group were pooled and a t-test was used. *p* values of <0.05 were considered as significant. Graphs were generated using Microsoft Office Excel 2007 version 14.0.

## 3. Results

### 3.1. Immune Response to Anti-HEV Vaccine Candidates in Monovalent and Combined Approaches

**Figure 1:**
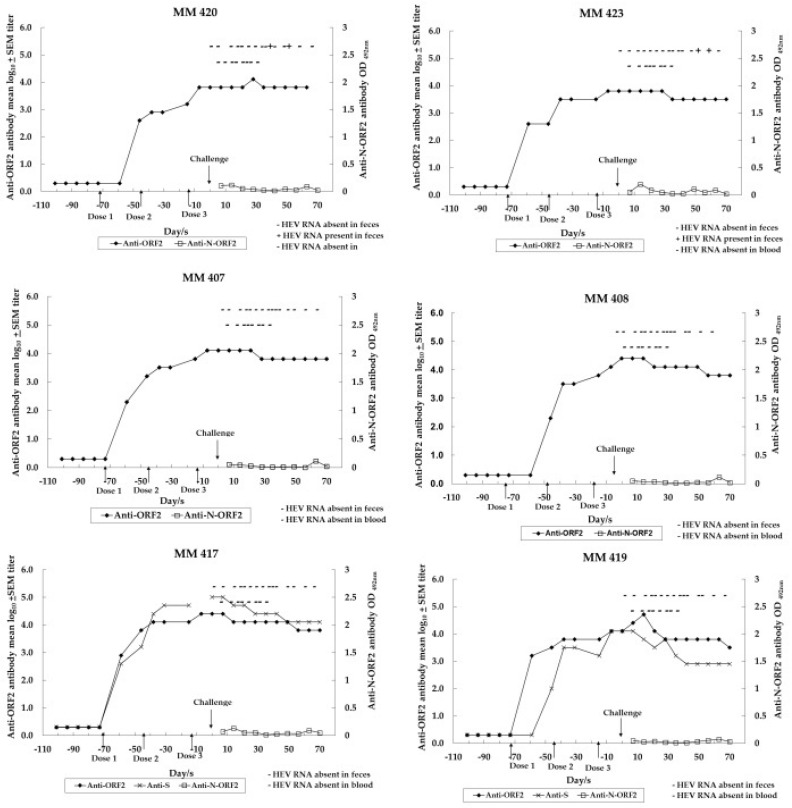
Homologous challenge of monkeys immunized with different vaccine candidates. Doses 1, 2, and 3 show immunization schedules. The downward arrow indicates an HEV challenge time-point (10^6^ HEV RNA copies; 10,000 MID_50_). Open squares (□) show anti-N-ORF2 antibody detection ELISA OD_492 nm_ values. Closed circles (•) show anti-ORF2 antibody log_10_ titers in the serum samples. Crosses (x) show anti-S antibody log_10_ titer in the serum samples (detection done in ELISA based on S protein obtained from SII, Pune, India). The presence or absence of HEV RNA in feces is marked by + or − signs (refer to the upper line). The presence or absence of HEV RNA in blood is marked by + or − signs (refer to the lower line). MM 420 and 423 were immunized with Lipo-NE-P, MM 407 and 408 were immunized with Lipo-NE-DP, and MM 417 and 419 received Lipo-NES-DP.

Four of the six monkeys seroconverted at 2 weeks following the first dose: MM 423 immunized with Lipo-NE-P (titer 400), MM 407 immunized with Lipo-NE-DP (titer 200), MM 417 and 419 both immunized with Lipo-NES-DP (titers 800 and 1600, respectively). For the two remaining monkeys: MM 420 immunized with Lipo-NE-P (titer 1600) and MM 408 immunized with Lipo-NE-DP (titer 6400) seroconverted at 4 weeks following the first dose (Figure 1).

Table 2 shows anti-ORF2 (NE) antibody reciprocal and geometric mean titers (GMTs)/mean log_10_ ± standard error of the mean (SEM) titers noted in monkeys belonging to the immunization and control groups following each of the three doses or at different time-points following the challenge (PC)/experimental infection (EI). The Anti-ORF2 antibody titers 2 weeks after dose 3 just before the challenge were 6400 (in both MM 420 and 423), 12,800 (in both MM 407 and 419), and 25,600 (in both MM 408 and 417) (Table 2).

**Figure 2:**
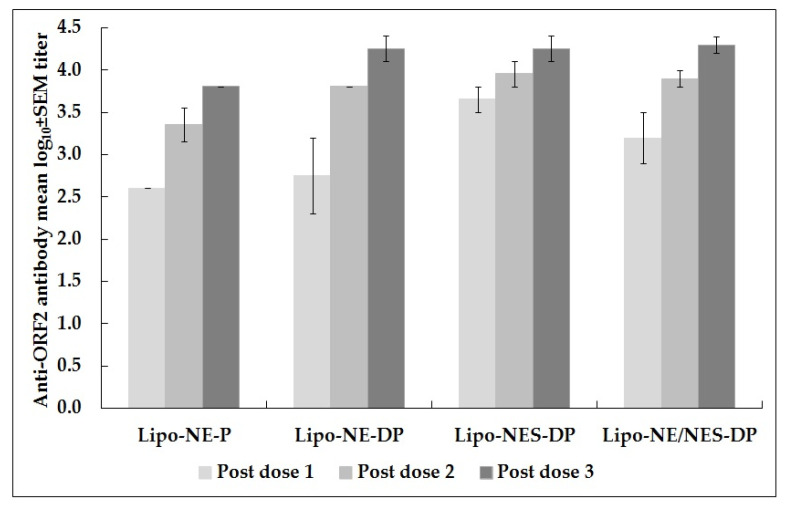
Anti-ORF2 antibody mean log_10_ titers in monkeys immunized with Lipo-NE-P (MM 420 and 423), Lipo-NE-DP (MM 407 and 408), and Lipo-NES-DP (MM 417 and 419) vaccine candidates. The last horizontal x axis data point (Lipo-NE/NES-DP) shows successive mean log_10_ titers following each dose calculated for the four monkeys (MM 407, 408, 417, and 419 immunized with Lipo-NE/NES-DP). Antibody titers were determined 4 weeks after each of dose 1, dose 2, and 2 weeks after dose 3. Error bars represent standard error of the mean (SEM) of log_10_ titer.

The Lipo-NES-DP group showed the highest anti-ORF2 antibody GMTs following both doses 1 (4525.5) and 2 (9051). Following the completion of the immunization schedule, anti-ORF2 antibody titers were equal in monkeys immunized with Lipo-NE-DP or Lipo-NES-DP (18,101.9) and were ~2.8 times higher than the titer (6400) achieved in monkeys immunized with Lipo-NE-P (*p* > 0.05). Following the completion of immunization schedule, anti-ORF2 antibody GMTs/mean log_10_ ± SEM calculated for four monkeys (MM 407, 408, 417, and 419) was 18,101.9/4.3 ± 0.1. Anti-ORF2 antibody titers increased successively as the immunization schedule progressed in each of the immunized monkeys (Table 2 and Figure 2).

Two weeks after dose 3 and just before the challenge, the anti-ORF2 antibody levels compared with the WHO HEV antibody reference, respectively, were 59.4 and 148.4 IU/mL in monkeys immunized with Lipo-NE-P, 177.0 and 240.8 IU/mL in monkeys immunized with Lipo-NE-DP, and 240.7 and 164.9 IU/mL in monkeys immunized with Lipo-NES-DP. Following the immunization/challenge, the anti-ORF2 IgG1 antibody was detected in all monkeys irrespective of the vaccine candidate administered, while the specific IgG2 and IgG4 antibodies were undetectable.

### 3.2. Immune Response to Anti-HBV Vaccine Candidate in Combined Approach

Two monkeys (MM 417 and 419) were immunized with the combined vaccine candidate (Lipo-NES-DP). MM 417 and MM 419 seroconverted to anti-HBV IgG antibodies at 2- and 4-weeks after dose 1, respectively (detection performed in ELISA based on S protein obtained from SIIPL). Table 3 shows the anti-HBV antibody levels induced in monkeys in mIU/mL. The anti-HBV antibody levels increased successively as the immunization schedule progressed in each of the immunized monkeys. Four weeks after dose 3- and 2-weeks after HEV challenge, the anti-HBV antibody levels in MM 417 and 419 were 89,703 and 8976 mIU/mL, respectively, and were higher than the corresponding levels (59,786 mIU/mL in MM 417 and 6213 mIU/mL in MM 419) achieved 2 weeks after dose 3 before the HEV challenge (Table 3). The HBV vaccine candidate in the combined formulation also induced a specific IgG1 antibody response in both the immunized monkeys after complete immunization and after HEV challenge. The anti-HBV IgG2 and IgG4 antibodies were undetectable.

### 3.3. Dynamics of HEV Infection in Control Monkeys

**Figure 3:**
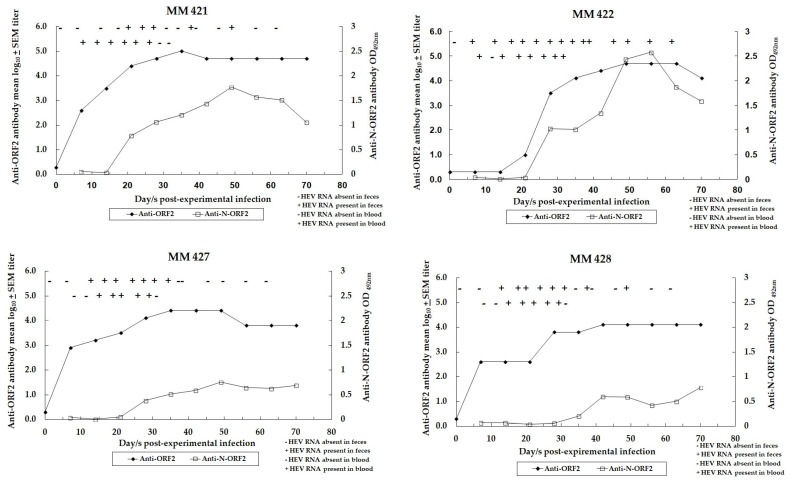
Dynamics of experimental HEV infection in control monkeys. On day zero, all the monkeys (MM 421, 422, 427, and 428) received intravenous inoculums of genotype 1 HEV (10^6^ HEV RNA copies). Open squares (□) show anti-N-ORF2 antibody detection ELISA OD_492 nm_ values. Closed circles (•) show anti-ORF2 antibody log_10_ titer in the serum samples. The presence or absence of the HEV RNA in feces is marked by + or − signs (refer to the upper line). The presence or absence of HEV RNA in the blood is marked by + or − signs (refer to the lower line).

Figure 3 depicts the dynamics of HEV infection in control and unimmunized monkeys (MM 421, 422, 427, and 428) experimentally infected with challenge inoculums of the virus (10^6^ HEV RNA copies, 10,000 MID_50_ HEV).

Two of four monkeys (MM 422 and 428) showed a moderate rise in serum ALT levels, with the peak values being 40 and 42 IU/liter on the 19th day following the experimental infection (Table 4).

All four control monkeys showed evidence of HEV infection as indicated by viremia, the excretion of the virus in feces, and the seroconversion to anti-HEV antibodies (anti-ORF2 and anti-N-ORF2). Post-experimental infection, viremia was evident in all four control monkeys and was detected from the eighth day onwards up to 1 month (Figure 3). HEV was excreted in the feces from the sixth day onwards up to 8 weeks post-experimental infection. The maximum viral load given as HEV RNA copies/mL in 10% (*w*/*v*) fecal suspension was detected at 17, 17, 17, and 20 days post-experimental infection in MM 421 (1.4 × 10^7^), MM 422 (8.7 × 10^8^), MM 427 (3.0 × 10^6^), and MM 428 (2.5 × 10^6^), respectively (Table 4).

Anti-N-ORF2 antibodies were detected in all control monkeys and were evident from 19 to 34 days up to 9 weeks post-experimental infection. Anti-ORF2 antibody titers in control monkeys that peaked from 5 to 7 weeks following experimental infection and the titers were 102,400 at 5 weeks in MM 421, 51,200 at 7 weeks in MM 422, 25,600 at 5 weeks in MM 427, and 12,800 at 6 weeks in MM 428 (Figure 3). Anti-ORF2 (NE) antibody GMTs/mean log_10_ ± SEM titers at 1 month, 11 months and 2 years 3 months post-experimental infection in control monkeys were 10,763.5/4.0 ± 0.3 (4525.5/3.7 ± 0.4), 3805.5/3.6 ± 0.3 (1131.4/3.1 ± 0.3), and 1345.4/3.1 ± 0.2, respectively (Table 2). Anti-ORF2 IgG subclass antibody was restricted to IgG1 type in all the control monkeys and the anti-ORF2 IgG2 and IgG4 antibodies were undetectable.

### 3.4. Assessment of HEV Infection in Challenged Monkeys

The protective efficacy of Lipo-NE-P, Lipo-NE-DP, and Lipo-NES-DP vaccine candidates was evaluated in the immunized monkeys post-challenge. Serum ALT levels did not rise in any of the immunized monkeys following challenge. However, in absence of a significant rise in the ALT levels in the unimmunized experimentally infected control monkeys, this parameter could not be used as evidence of lack of hepatitis in immunized monkeys (Table 4). Viremia and seroconversion to the N-ORF2 region of ORF2 protein was not detected in any of the immunized monkeys (Figure 1). Complete protection from infection was noted in the monkeys immunized with Lipo-NE-DP (MM 407 and 408) and Lipo-NES-DP (MM 417 and 419) vaccine candidates, as none of the monkeys excreted HEV in their feces (Table 4). Monkeys immunized with the Lipo-NE-P (MM 420 and 423) vaccine candidate showed the transient excretion of HEV in feces. HEV RNA (copies/mL) was detected in 10% (*w*/*v*) fecal suspensions of MM 420 on days 37 (9.7 × 10^5^) and 49 (2.8 × 10^5^). Similarly, MM 423 excreted HEV in feces on days 49 (1.0 × 10^5^) and 56 (3.2 × 10^3^) post-challenge. The difference in the mean log_10_ of the viral load in the feces of the control and Lipo-NE-P group monkeys was 1.1 (*p* > 0.05).

**Figure 4:**
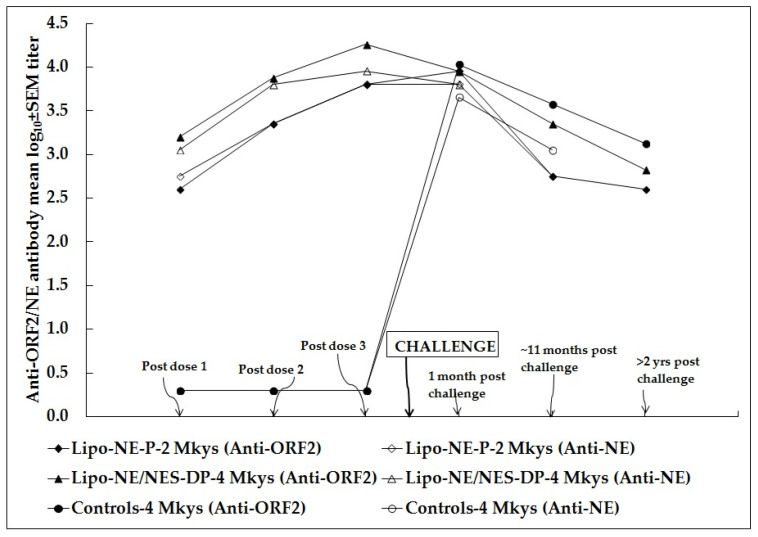
Kinetics of anti-ORF2/NE antibody mean log_10_ titers at cardinal time-points in monkeys immunized with Lipo-NE-P (MM 420 and 423; *n* = 2) and Lipo-NE/NES-DP (MM 407, 408, 417, 419; *n* = 4) vaccine candidates. The anti-ORF2/NE antibody titers are also shown for four control monkeys that were experimentally infected (MM 421, 422, 427, and 428). Antibody titers were determined 4 weeks after each of dose 1 and dose 2; 2 weeks after dose 3, just before the challenge; and 1 month; ~11 months; >2 years post-challenge (PC)/experimental infection (EI). The log_10_ titer ± standard error of the mean (SEM) has been given in Table 2.

At 1 month, 11 months, and 2 years and 3 months following challenge, the anti-ORF2 (NE) antibody GMTs/mean log_10_ ± SEM titers in monkeys immunized with Lipo-NE-P were 9051.0/4.0 ± 0.2 (6400.0/3.8 ± 0.0), 565.7/2.8 ± 0.5 (565.7/2.8 ± 0.2) and 400.0/2.6 ± 0.3, respectively. At the same post-challenge time-points, the anti-ORF2 (NE) antibody GMTs/mean log_10_ ± SEM titers in monkeys immunized with Lipo-NE-DP and Lipo-NES-DP were 9051/4.0 ± 0.2 (6400/3.8 ± 0.0), 1600/3.2 ± 0.0 (1131.4/3.1 ± 0.5), 565.7/2.8 ± 0.2, and 9051/4.0 ± 0.2 (6400/3.8 ± 0.3), 3200/3.5 ± 0.3, 800/2.9 ± 0.3, respectively. Similarly, the anti-ORF2 (NE) antibody GMTs/mean log_10_ ± SEM titers calculated for four monkeys representing two immunization groups were 9051.0/4.0 ± 0.1 (6400.0/3.8 ± 0.1), 2262.7/3.4 ± 0.2 and 672.7/2.8 ± 0.1, respectively (Table 2). The kinetics of the anti-ORF2/NE antibody mean log_10_ titers at cardinal time-points in monkeys immunized with Lipo-NE-P (MM 420 and 423) and Lipo-NE/NES-DP (MM 407, 408, 417, and 419) vaccine candidates along with experimentally infected control monkeys (MM 421, 422, 427, and 428) are shown in Figure 4.

Anti-HBV antibody levels declined successively until >2 years post-HEV challenge in each monkey (MM 417 and 419) immunized with combined vaccine candidate Lipo-NES-DP (Table 3).

## 4. Discussion

In continuation with our earlier study, the present study reports the immunogenicity of our promising hepatitis E monovalent vaccine candidates and of a combined HEV + HBV vaccine candidate that retained high immunogenicity against both components [10]. Of note, we used just the neutralizing epitopes region of HEV in its DNA and/or protein forms (150 amino acids) against the ORF2 proteins assessed in clinical trials, rHEV, HEV 239, and p179 [19,20,21,22,23].

Hepatitis E is a major public health problem in India presenting both as an epidemic and as a sporadic disease. Severity and high mortality among pregnant women characterized this disease and the development of a vaccine for this vulnerable group is essential. Additionally, HBV and HCV carriers also need to be immunized to reduce the severity/mortality following exposure to HEV [4,5,16]. For this, the carrier status must be known for different populations like rural, tribal, and those residing in difficult-to-reach areas. This vaccine may not be economically viable, but it is important. Since this is an important issue and extensive work on this virus and disease has previously been performed by our laboratory, vaccine development became the next priority. We would like to add here that, during a large epidemic of hepatitis E in Karad, we assessed the utility of passive immune prophylaxis in pregnant women using normal human immunoglobulin (NHIG). However, there was a loss to follow-up as most pregnant women who received the NHIG traveled out of the study area [24]. As anti-HEV positivity in adults was ~40%, the immunoglobulins did have anti-HEV activity at low titers. We did find a benefit of this treatment and recommended the development of immunoglobulins from high-tittered blood donors. This study provided evidence of the usefulness of antibodies in protection and prompted us to develop a vaccine eliciting high anti-HEV titers.

There was another important issue. It was being debated that anti-HEV antibodies decline sharply after natural HEV infection. Therefore, we explored the use of immunogen in its DNA and the corresponding protein forms formulated in liposome as a possible candidate eliciting high titers. At that time, the use of DNA in a vaccine required long-term experiments to ensure safety. It is extremely important to note that, during the recent pandemic, the COVID-19 DNA vaccine was developed, produced, and approved by the regulatory authorities for human use [25]. Unfortunately, it was not so simple then. To address this difficulty, we also evaluated the liposome-rNEp formulation.

As far as the monovalent HEV vaccine candidate is concerned, the NE protein, alone (Lipo-NE-P) or with the corresponding DNA (Lipo-NE-DP), induced comparable levels of anti-HEV antibodies in monkeys (*p* > 0.05) in the present study. Following the challenge, the absence of viremia, the maintenance of baseline ALT levels, and a lack of significant rise in anti-HEV antibody titers were documented in each of the immunized monkeys from both vaccine groups. Remarkably, the anti-N-ORF2 antibodies suggestive of the active replication of HEV were absent. The monkeys immunized with NE DNA + corresponding rProtein (Lipo-NE-DP) did not support the HEV replication following the challenge (Figure 1). Yet, at this point, rNEp appeared to be a better and more acceptable vaccine candidate. Nevertheless, the inclusion of the NE DNA component in the vaccine candidate certainly improved its efficacy by providing complete protection from infection as evidenced by the lack of HEV excretion in post-challenge feces, confirming our previous observations [10]. In our earlier study, two doses of liposome-complexed NE DNA and corresponding protein elicited sterilizing immunity. A two-dose regimen of liposome-complexed NE DNA plus protein was found to be optimum and the third dose was not necessary (*p* > 0.05 for both comparisons) (Figure 2). However, to achieve a similar kind of complete protection using only protein-based vaccine candidate, the requirement of an additional dose was contemplated. Hence, a total of three doses of each of the vaccine candidates was administered to the monkeys. Yet, the vaccine candidate of rNE protein alone provided partial protection as the transient excretion of a low HEV viral load in the post-challenge feces of immunized monkeys was evident (Table 4). It seems plausible that, by increasing the rNE protein dose concentration to 30–50 µg, complete protection can be achieved, but it needs to be proved experimentally. Of note, a sharper decline in anti-HEV antibody titers was noticed in the monkeys immunized with DNA-containing formulations (Lipo-NE/NES-DP) during the follow-up >2 years after the challenge. Surprisingly, the antibodies elicited by the vaccine candidate with NE protein alone were more stable over the same time (Figure 4). Thus, the liposome-complexed NE protein alone undoubtedly holds promising candidature as a more feasible vaccine. While dispensing of the DNA component, it has achieved partial protection from infection and has curbed systemic infection after the initial intravenous challenge. We used liposomes as an adjuvant instead of alum typically used in the other formulations [19,20,21,22,23]. It is of major significance that the immunized-challenged monkeys were followed for ~2 years to provide long-term satisfactory results. The reason that the early higher antibody response was followed by a rapid decline induced by DNA-containing vaccine candidates remains unclear and requires further studies. In situations needing an early higher antibody response, the feasibility of using DNA-containing vaccines needs further evaluation.

Our next aim was the development of a combined (E + B) vaccine employing a similar approach. Despite the global use of the protein-based hepatitis B vaccine and associated success stories, we explored the use of the DNA component since the target population, namely pregnant women and HCV carriers, are immunocompromised. For this specific population, the evaluation of vaccine candidates with superior immunogenicity was considered to be worth the efforts. While both vaccines can be given individually to the high-risk populations, enhanced immunity against the HBV component did suggest that this could be one of the useful strategies. The constant availability of both vaccines at all the immunization centers is another concern. Although the population requiring combined vaccine appears to be low, the use of individual vaccines may be a more feasible approach; a greater number of injections will also need to be administered and immunity against HBV vaccine may be inferior.

The combined vaccine candidate (Lipo-NES-DP) induced high anti-HBV antibody levels in both immunized monkeys. Surprisingly, 2 weeks after HEV challenge, the anti-HBV antibody levels increased in both immunized monkeys (Table 3). In the absence of a control group immunized with the rS protein and corresponding DNA, it is difficult to conclude whether the enhancement of anti-HB titers during the next two weeks was a natural pattern or effect of HEV infection. With the definition of protective anti-HBs levels as ≥10 mIU/mL, the HBV-specific component in the combined vaccine candidate induced approximately 600–6000 times higher anti-HBs levels [26]. One of the monkeys (MM 419) showed anti-HBs levels of 1760 mIU/mL approximately more than 2 years after immunization. Irrespective of the type of immunogen, HEV/HBV-specific IgG1 subclass antibody was detected in all the immunized monkeys, which is suggestive of Th2 immune response.

The HEV-specific response induced by the combined (Lipo-NES-DP) and monovalent (Lipo-NE-DP) vaccine candidates having DNA components (NE and/or S) was comparable in terms of the anti-HEV antibody titers achieved after the completion of the immunization schedule (*p* > 0.05). The HBV component appeared to positively enhance the anti-HEV antibody titers following doses 1 and 2 in monkeys immunized with the combined vaccine candidate compared to monkeys immunized with Lipo-NE-DP. The mechanism of (the additional adjuvant effect?) such an enhancement needs to be studied in detail. Post-challenge, each of the immunized monkeys from the combined vaccine group also lacked evidence for systemic virus replication and excretion into the feces. Notably, the sterilizing immunity documented in our previous monkey experiment was reproduced not just by the monovalent hepatitis E vaccine candidate (Lipo-NE-DP) but also by the combined vaccine candidate (Lipo-NES-DP) [10].

Despite using a homologous HEV challenge dose (10,000 MID_50_) that was 100 times higher than that used in our earlier monkey experiment (100 MID_50_), all the monkeys immunized with vaccine candidates containing both NE DNA and protein were protected from infection, as evidenced by the absence of HEV in feces [10]. Although rhesus monkeys are used in HEV research, ALT rise was not uniformly observed and remains less pronounced and transient whenever present. This was evidenced in the non-immunized monkeys infected with the virus. Taken together, we could not use ALT as a marker for hepatitis in HEV-infected monkeys but needed to depend on virus replication. The lack of severe disease/mortality in HEV-infected pregnant monkeys is the most significant observation supporting the lack of uniform ALT rise [27].

With the availability of standard antibody preparation by WHO (100 IU/mL), it is now possible to compare the results from different studies. In the NIH (US) study, complete protection against hepatitis was achieved with anti-HEV levels of approximately 64–80 IU/mL in rhesus monkeys, although protection against infection was partial as evidenced by viremia. Anti-HEV levels of ≥175 IU/mL protect against both hepatitis and infection [19]. The HEV 239 vaccine when administered to rhesus monkeys showed antibody levels ranging between 186 and 2504 IU/mL in all the vaccinated animals before the challenge with the virus. This vaccine provided complete protection when challenged with the 10^4^ HEV genomic dose while 75% protection was observed at a higher challenge virus (10^7^ HEV genomic dose) [20]. The precise protective anti-HEV antibody levels could not be documented during the pre-licensure clinical testing of the HEV 239 vaccine in China as the vaccine group lacked hepatitis E cases [22]. During the phase III trial, 1 month after immunization, a peak antibody level of 15 IU/mL (geometric mean concentration) was induced in participants that were seronegative at baseline. An antibody level of 0.6 WHO IU/mL was found in persons who recovered from a natural infection, whereas an antibody level of 80.9 WHO IU/mL was induced during acute hepatitis E [28]. In our study, the anti-HEV levels ranged from 59.4 to 240.8 IU/mL post-dose 3. Based on the results, it seems possible that the NE protein-based vaccine with a 30–50 μg dose could be the best alternative for a monovalent HEV vaccine. In our earlier monkey experiment, the anti-NE titer of 200 appeared to protect against disease [10]. Since HEV is transmitted feco-orally in a natural setting, it is plausible that lower anti-HEV antibody levels may be more protective than those achieved in the present study wherein monkeys were intravenously challenged with a large viral dose. Of note, the HEV-specific long-lived antibodies and memory B cells were observed to be maintained for several years in hepatitis E recovered individuals [29].

Our study has certain limitations. Rhesus monkeys do not always show a rise in serum ALT levels following virus inoculation. Hence, the comparison of liver damage cannot be made based on this marker. Because DNA vaccines were not immunogenic in mice, we did not receive permission to evaluate these in monkeys. Moreover, we did not include the liposome-complexed S protein alone group, though it was used in mice [11]. Finally, we did not address cellular immunity to HEV and HBV.

In conclusion, we explored a novel approach for developing monovalent hepatitis E vaccine candidates using liposome-complexed NE DNA and/or the corresponding protein and a combined vaccine candidate for hepatitis E and B to be used in high-risk populations. However, due to the presence of DNA components and economic viability, these vaccine candidates did not progress further. Although DNA vaccines are accepted now, the high efficacy of the NE protein-based vaccine candidate makes it the priority one. The long-term persistence of antibodies in humans following only protein or with DNA vaccines needs careful evaluation. The absence of liposome-formulated NE + S proteins remains a major drawback and should be studied as soon as possible.

## Figures and Tables

**Table 1:** Details of vaccine candidates formulated in liposomes or empty liposomes used for monkey immunizations.

Monkey ID No.	Vaccine Candidate Formulations in Liposomes or Empty Liposomes	Challenged/Experimentally Infected (HEV RNA Copies/mL)
MM 420, 423	Lipo-NE-P (Protein of HEV)	Yes (10^6^)
MM 407, 408	Lipo-NE-DP (DNA + Protein of HEV)	Yes (10^6^)
MM 417, 419	Lipo-NES-DP (DNA + Protein of each of HEV and HBV)	Yes (10^6^)
Unimmunized Controls MM 421, 422, 427, 428	PBS with empty liposomes	Yes (10^6^)

**Table 2:** Anti-ORF2 (NE) antibody reciprocal and GMTs/mean log_10_ ± SEM titers in immunized and control monkeys.

Groups	Monkey ID No.	Anti-ORF2 (NE) Antibody Reciprocal and (GMTs/Mean log_10_ ± SEM) Titers					
		Post-Dose 1	Post-Dose 2	Post-Dose 3	1 Month PC/EI **	~11 Months PC/EI	~2 Years 3 Months PC/EI
Control	MM 421	NA *	NA	NA	51,200 (12,800)	12,800 (6400)	3200
	MM 422	NA	NA	NA	3200 (400)	6400 (400)	3200
	MM 427	NA	NA	NA	12,800 (12,800)	800 (800)	800
	MM 428	NA	NA	NA	6400 (6400)	3200 (800)	400
		NA	NA	NA	10,763.5/4.0 ± 0.3 (4525.5/3.7 ± 0.4)	3805.5/3.6 ± 0.3 (1131.4/3.1 ± 0.3)	1345.4/3.1 ± 0.2
Lipo-NE-P	MM 420	400 (800)	1600 (1600)	6400 (6400)	12,800 (6400)	200 (400)	200
	MM 423	400 (400)	3200 (3200)	6400 (6400)	6400 (6400)	1600 (800)	800
		400/2.6 ± 0.0 (565.7/2.8 ± 0.2)	2262.7/3.4 ± 0.2 (2262.7/3.4 ± 0.2)	6400/3.8 ± 0.0 (6400/3.8 ± 0.0)	9051/4.0 ± 0.2 (6400/3.8 ± 0.0)	565.7/2.8 ± 0.5 (565.7/2.8 ± 0.2)	400/2.6 ± 0.3
Lipo-NE-DP	MM 407	1600 (800)	6400 (6400)	12,800 (6400)	6400 (6400)	1600 (3200)	800
	MM 408	200 (800)	6400 (6400)	25,600 (12,800)	12,800 (6400)	1600 (400)	400
		565.7/2.8 ± 0.5 (800/2.9 ± 0.0)	6400/3.8 ± 0.0 (6400/3.8 ± 0.0)	18,101.9/4.3 ± 0.2 (6400/3.8 ± 0.0)	9051/4.0 ± 0.2 (6400/3.8 ± 0.0)	1600/3.2 ± 0.0 (1131.4/3.1 ± 0.5)	565.7/2.8 ± 0.2
Lipo-NES-DP	MM 417	6400 (1600)	12,800 (6400)	25,600 (12,800)	12,800 (12,800)	6400	1600
	MM 419	3200 (1600)	6400 (6400)	12,800 (6400)	6400 (3200)	1600	400
		4525.5/3.7 ± 0.2 (1600/3.2 ± 0.0)	9051/4.0 ± 0.2 (6400/3.8 ± 0.0)	18,101.9/4.3 ± 0.2 (9051/4.0 ± 0.2)	9051/4.0 ± 0.2 (6400/3.8 ± 0.3)	3200/3.5 ± 0.3	800/2.9 ± 0.3
Lipo-NE/NES-DP	MM 407	1600 (800)	6400 (6400)	12,800 (6400)	6400 (6400)	1600 (3200)	800
	MM 408	200 (800)	6400 (6400)	25,600 (12,800)	12,800 (6400)	1600 (400)	400
	MM 417	6400 (1600)	12,800 (6400)	25,600 (12,800)	12,800 (12,800)	6400	1600
	MM 419	3200 (1600)	6400 (6400)	12,800 (6400)	6400 (3200)	1600	400
		1600.0/3.2 ± 0.3 (1131.4/3.1 ± 0.1)	7610.9/3.9 ± 0.1 (6400/3.8 ± 0.0)	18,101.9/4.3 ± 0.1 (7610.9/3.9 ± 0.1)	9051/4.0 ± 0.1 (6400/3.8 ± 0.1)	2262.7/3.4 ± 0.2	672.7/2.8 ± 0.1

* NA—not applicable; ** PC/EI—post-challenge/experimental infection.

**Table 3:** Anti-S antibody levels in immunized monkeys.

Group	Monkey ID No.	Anti-HBV Antibody Levels in mIU/mL					
		4 Weeks Post-Dose 1	4 Weeks Post-Dose 2	2 Weeks Post-Dose 3 before HEV Challenge	4 Weeks Post-Dose 3, 2 Weeks Post HEV Challenge	~11 Months, 2 Weeks Post-Dose 3	~2 Years 4 Months Post-Dose 3
Lipo-NES-DP	MM417	130	7559	59,786	89,703	26,767	-
	MM419	6	406	6213	8967	8120	1760

**Table 4:** Summary of challenge/experimental infection experiment.

Groups	Monkey ID No.	Anti-ORF2 Antibody Titer after Dose 3 before Challenge/5 Weeks Post EI	Peak/Pre-Challenge Ratio of ALT Values (Day after Challenge/EI)	HEV RNA Copies in Feces, Peak Titer/mL in 10% (*w*/*v*) Fecal Suspension (Day PC/EI **)
Lipo-NE-P	MM 420	6400	1.0 (12th)	9.7 × 10^5^ (37th)
	MM 423	6400	1.1 (12th)	1.0 × 10^5^ (49th)
Lipo-NE-DP	MM 407	12,800	0.9 (12th)	ND ^a^
	MM 408	25,600	0.9 (12th)	“
Lipo-NES-DP	MM 417	25,600	1.1 (12th)	“
	MM 419	12,800	1.0 (12th)	“
Control	MM 421	102,400	1.2 (12th)	1.4 × 10^7^ (17th)
	MM 422	12,800	1.9 (19th)	8.7 × 10^8^ (17th)
	MM 427	25,600	0.8 (22nd)	3.0 × 10^6^ (17th)
	MM 428	6400	1.7 (19th)	2.5 × 10^6^ (20th)

^a^ ND—not detected; ** PC/EI—post-challenge/experimental infection.

## Data Availability

All datasets generated from this study are included in the article.
